# CSE/H_2_S ameliorates colitis in mice *via* protection of enteric glial cells and inhibition of the RhoA/ROCK pathway

**DOI:** 10.3389/fimmu.2022.966881

**Published:** 2022-09-15

**Authors:** Song Wang, Yanyu Ding, Wenjun Jiang

**Affiliations:** ^1^ Department of Gastroenterology, The First Affiliated Hospital of USTC, Division of Life Sciences and Medicine, University of Science and Technology of China, Hefei, China; ^2^ Department of Pharmacology, School of Basic Medical Sciences, Anhui Medical University, Hefei, China

**Keywords:** hydrogen sulfide, cystathionine gamma-lyase, enteric nervous system, rho-associated kinases, colitis, animal model

## Abstract

The enteric glial cells (EGCs) participate in the homeostasis of the gastrointestinal tract, and RhoA/ROCK signaling pathway plays a vital role in colonic tight junctions. Hydrogen sulfide (H_2_S) has been reported to alleviate colitis. However, the effect and mechanism of endogenous H_2_S on colitis remain unclear. This study established a Cystathionine-γ-lyase (CSE) knockout mouse model, a significant source of H_2_S production in the gut. The role of CSE-produced H_2_S on EGCs and the RhoA/ROCK signaling pathway was investigated in experimental colitis using CSE knockout (KO) and wild-type (WT) mice. CSE gene knockout animals presented with disease progression, more deteriorated clinical scores, colon shortening, and histological damage. EGCs dysfunction, characterized by decreased expression of the glial fibrillary acidic protein (GFAP), C3, and S100A10, was observed in the colon of WT and KO mice, especially in KO mice. RhoA/ROCK pathway was significantly upregulated in colon of colitis mice, which was more evident in KO mice. Pretreatment with NaHS, an exogenous H_2_S donor, significantly ameliorated mucosal injury and inhibited the expression of proinflammatory factors. Furthermore, we found that NaHS promoted the transformation of EGCs from “A1” to “A2” type, with decreased expression of C3 and increased expression of S100A10. These findings suggest that CSE/H_2_S protects mice from colon inflammation, which may be associated with preserving EGCs function by promoting EGCs transformation and inhibiting the RhoA/ROCK pathway.

## Introduction

Inflammatory bowel diseases (IBDs) are chronic progressive and unpredictable inflammatory diseases of gastroenteric tissue, which primarily include Crohn’s disease and ulcerative colitis (UC) (1). Between 1990 and 2017, China experienced an increase in the age-standardized rate of prevalence, incidence, and the years of life lived with disability of IBD (2). The clinical manifestations of UC include diarrhea, abdominal pain, weight loss, and bloody stools, where sustained remission is not currently achievable (3). Although there have been many studies on UC, its pathological mechanism remains poorly understood. Previous immunological and genetic studies have demonstrated that the inflammation in UC patients was closely associated with a burst of cytokines or/and chemokines production from immune cells and epithelial cells, which was linked to the dysfunction of intestinal epithelial barrier (IEB) homeostasis (3, 4).

Enteric neurons and glial cells (EGCs) play a significant role in maintaining IEB function. However, enteric neurons alone couldn’t maintain normal function, they are part of enteric nervous system and brain-gut axis, and EGCs are found accompanying enteric neurons throughout the gut (5). EGCs contribute to restoring the integrity of injured epithelium in IBD, and the ablation of EGCs leads to increased IEB permeability and intestinal inflammation (6, 7). EGCs present abnormalities in UC. EGCs dysfunction, manifested by the decrease of GFAP, was observed in dextran sodium sulfate (DSS)-induced murine UC (4). Neurotrophic growth factors can stabilize the intestinal barrier by preventing the apoptosis of EGCs and enterocytes (8).

Hydrogen sulfide (H_2_S) is a ubiquitous second messenger molecule. H_2_S is considered the third gasotransmitter, in addition to nitric oxide (NO) and carbon monoxide (CO). It is involved in inflammation, gut motility, oxidative stress, ulcer healing, vascular tone, neuromodulation, cryoprotection, memory formation, hormone secretion, apoptosis, and other vital biologic functions (9). H_2_S in the gut is produced by the enzymes cystathionine-β-synthase (CBS) and Cystathionine-γ-lyase (CSE) of the host and sulfate-reducing bacteria (SRB) of intestinal resident microbes (9–11). CBS is the main source of H_2_S in the central nervous system and CSE is the major source in the peripheral system (12). Grasa et al. found the interaction between TLR2 and TLR4 and the sulfide system in regulating colonic motility (11). However, detailed interactions of H_2_S with other signaling mediator has not yet been elucidated. The RhoA/ROCK signaling pathway is related to UC (13). Inhibiting the RhoA/ROCK pathway might be a new approach in treating UC (14, 15). Moreover, we previously found that exogenous H_2_S protects colon in murine UC by inhibiting the RhoA/ROCK pathway (16). CSE-derived H_2_S inhibited reactive astrocyte proliferation and promoted functional neural recovery after cerebral ischemia/reperfusion injury in mice *via* the RhoA/ROCK_2_ pathway (17). However, the effect and mechanism of endogenous H_2_S on the EGCs function and RhoA/ROCK signaling pathway following colitis are still unclear.

Considering the crucial role of EGCs function and RhoA/ROCK signaling pathway, as well as the potential therapeutic effect of H_2_S in UC, this study was to investigate whether CSE-derived H_2_S could alleviate DSS-induced acute intestinal inflammation by improving the recovery of EGCs dysfunction and inhibiting inflammation through targeted down-regulation of the RhoA/ROCK signaling pathway.

## Materials and methods

### Reagents

NaHS was purchased from Sigma Aldrich (St. Louis, USA); Dextran sulfate sodium (DSS, 36–50kDa) was purchased from Shanghai Yi Sheng Biological Company (Shanghai, China); Primary CSE, glial fibrillary acidic protein (GFAP), C3, S100 calcium-binding protein A10 (S100A10), RhoA, ROCK_1_, and ROCK_2_ antibodies were obtained from Abcam (San Francisco, CA, USA); Horseradish peroxidase-conjugated secondary antibodies were obtained from Santa Cruz Biotechnology (Santa Cruz, CA, USA). RhoA, ROCK_1_, ROCK_2_, Interleukin-6 (IL-6), H_2_S, and tumor necrosis factor-α (TNF-α) test kits were obtained from Jiangsu Meimian Biological Company (Nanjing, China).

### Experimental animals

Adult (6−8weeks) CSE knockout (KO) and wild-type (WT) C57BL/6 mice weighing 20 to 24g were supplied by Shanghai Bio model Organism Science & Technology Development Co., Ltd.; all animals were kept in-house, housed with a 12-h light/dark cycle per day and constant temperature (22 ± 2°C) and relative humidity (55 ± 5%). The mice were raised to adapt to the new environment for one week before the experiment. All animals have free access to a standard diet and water ad libitum. The program was approved by the Animal Experimental Ethics Review Committee of the University of Science and Technology of China under protocol number 2019-N(A)-196).

### Experimental design and drug treatment

Thirty-six mice were randomly divided into six groups (n = 6). Two groups (WT control and KO control) received sterilized tap water ad libitum, and the other animals received 3% DSS dissolved in drinking water for seven days to induce colitis. Two groups (WT DSS+NaHS and KO DSS+NaHS) received NaHS at 4.8mg/kg by intraperitoneal injection, as previously reported (16, 17). On the 7th day, all mice were sacrificed 12 h later after the last administration. The length of the colons was measured and then washed instantly using ice-cold physiological saline. One part of colon was rapidly divided and fixed in 10% formalin for pathological examination, and the remaining parts were stored at −80°C for western blotting assay and immunofluorescence.

### Disease activity index

The severity of colitis was assessed by monitoring the disease activity index (DAI) during acute colitis, including body weight loss, stool consistency, and fecal bleeding. The DAI was calculated as described previously (18). The difference between the initial and testing weights was calculated as weight loss. Fecal bleeding was tested using a fecal occult blood test kit—two independent observers who were blinded to the experimental conditions accumulated the DAI scores.

### Histological examination

The mouse colon sections were fixed in 4% phosphate-buffered saline (PBS)-buffered formaldehyde for 48 h. Then, the samples were dehydrated, embedded in paraffin, and cut into 4-μm fractions. The sections were stained with hematoxylin and eosin (H&E) and observed under light microscopy. Histological scores were calculated by two pathologists uninformed of the experimental grouping. Each sample was graded semi-quantitatively from 0 to 3 based on four criteria: (1) leucocyte infiltration in the lamina propria; (2) degree of epithelial hyperplasia and goblet cell depletion; (3) area of tissue affected; and (4) presence of markers of severe inflammation such as crypt abscesses, submucosal inflammation, and ulcers (19).

### ELISA assays of inflammatory cytokine expression

After the behavior experiment, the mice were killed under deep anesthesia, and the sera were collected. The colon was cut into small pieces and homogenized with iced cold Tris-HCl buffer to extract total protein. The expression levels of cytokines IL-6 (Cat No.2101M08) and TNF-α(Cat No.2101M26) in the serum of mice and the RhoA(Cat No.2108M36), ROCK_1_(Cat No.2108M24), ROCK_2_(Cat No.2108M41), and H_2_S(Cat No.2109M33) contents in the colon were measured by enzyme-linked immunosorbent assay (ELISA) kits (Jiangsu Meimian Biological Company). All procedures followed the manufacturer’s guidelines. IL-6 and TNF-α were expressed as pg/ml, and the RhoA, ROCK_1_, ROCK_2_, and H_2_S content was expressed as ng/ml, IU/L, IU/L, and nmol/g protein, respectively. The intra-assay coefficient of variation (CV) and inter-assay CV were less than 10% and 15%, respectively.

### Western blotting analysis

As described previously, the total protein was extracted from colon tissues after the mice were decapitated under deep anesthesia and quantified using a protein assay kit (Beyotime Biotechnology, Shanghai, China) (16). Equal amounts of proteins were loaded and separated by electrophoresis and then transferred onto polyvinylidene difluoride membranes. The membranes were blocked with 5% defatted milk in Tris-buffered saline with 0.05% Tween 20 for one h at room temperature and then incubated with primary antibodies against RhoA, ROCK_1_, ROCK_2_, GFAP, S100A10, C3, or GAPDH overnight at 4°C. The catalog numbers and dilutions of each antibody are shown in **Table 1**. After incubation with the corresponding secondary antibody (1: 5000) for one h at room temperature, the membranes were visualized with an ECL (enhanced chemiluminescence) kit (Thermo Fisher Scientific, Massachusetts, USA) and scanned with a Chemi Q4800 mini imaging system (Shanghai Bioshine Technology, Shanghai, China). The relative expression levels of proteins were corrected by GAPDH.

**Table 1:** Antibodies used in the study.

Antibody	Company	Order Number	WB	IF
Anti-GFAP	Affinity, China	GB12096	1:2000	1:200
Anti-S100a10	Abcam, USA	Ab76472	1:5000	1:100
Anti-C3	Abcam, USA	Ab200999	1:2000	1:100
Anti-RhoA	Abcam, USA	Ab187027	1:5000	
Anti-ROCK_1_	Abcam, USA	Ab134181	1:5000	
Anti-ROCK_2_	Abcam, USA	Ab125025	1:5000	
Anti-NeuN	Abcam, USA	Ab279296		1:100
Anti-MBP	Santa Cruz, USA	sc271524		1:100
Anti-GAPDH	Affinity, China	Ab-AF7021	1:5000	
Peroxidase-Conjugated Goat anti-Rabbit IgG(H+L)	Zhongshan Jinqiao, China	ZB-2301	1:5000	
Peroxidase-Conjugated Goat anti-Mouse IgG(H+L)	Zhongshan Jinqiao, China	ZB-2305	1:5000	
Goat Anti-Mouse IgG (H&L) CY3	Boster Biological Technology, China	BA-1031		1:200
Goat Anti-Rabbit IgG (H&L) FITC	Boster Biological Technology, China	BA-1105		1:200

### Immunofluorescence

Paraffin-embedded colon sections were deparaffinized in xylene and rehydrated through graded alcohol to water. After unmasking antigens by 0.01 mol/L citrate buffer solution, the colon sections were blocked with 2% BSA and stained with anti-Myelin basic protein (MBP) (Santa Cruz, USA), anti-Neuronal Nuclei (NeuN)(Abcam), and anti-GFAP (Abcam), anti-C3 (Abcam), and anti-S100A10(Abcam) primary antibodies overnight at 4°C. Signals were determined using FITC-conjugated secondary antibodies (Boster Biological Technology) and then counterstained with 4’,6-diamidino-2-phenylindole (DAPI) (Abcam). The catalog numbers and dilutions of each antibody are shown in **Table 1**. Images were collected on a Leica TCS SPS microscope (Wetzlar, Germany), for fluorescence microscopy was used for nuclear counterstaining. ImageJ software was used to analyze fluorescence intensity in colon sections statistically.

### Statistical analysis

Data were analyzed using SPSS 25.0 (IBM, Armonk, NY, USA) software. GraphPad Prism 5.0 (GraphPad; San Diego, CA, USA) was used for statistical analysis. Results are shown as the mean ± S.E.M. The mean between groups was analyzed by one-way ANOVA and Fisher’s LSD test. P<0.05 was considered significant.

## Results

### Effect of CSE-derived H_2_S on the colonic mucosa in the DSS-induced ulcerative colitis animal model

**Figure 1:**
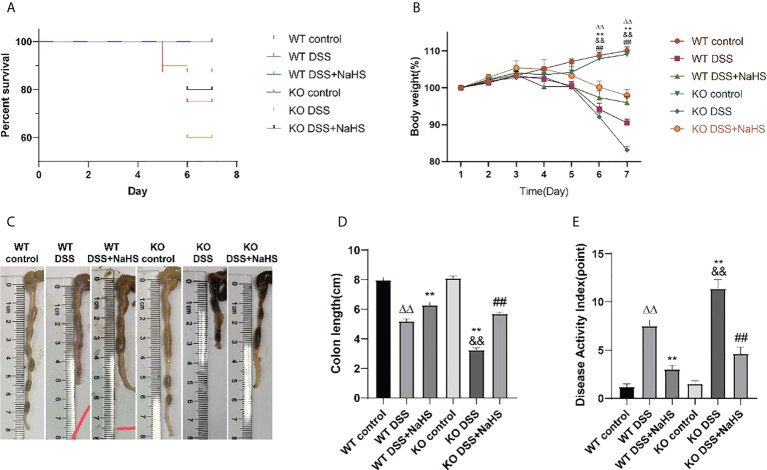
Effect of cystathionine-γ-lyase (CSE)-derived hydrogen sulfide (H2S) on dextran sodium sulfate (DSS)-induced ulcerative colitis in mice. **(A)** Percent survival. **(B)** Body weight (shown as the percentage of initial body weight). **(C)** Typical pictures of colon. **(D)** Colon length. **(E)** Disease activity index (DAI) score. All data are expressed as the mean ± SEM, N=6. ΔΔP 0.01 vs wild-type(WT) control group; **P < 0.01 vs WT DSS group; ^&&^P < 0.01 vs knockout (KO) control group; ^##^P < 0.01 vs KO DSS group.

All control mice did not die in the experimental modeling process, and the DSS modeling mortality of CSE knockout mice was higher than that of normal mice. However, the mortality of DSS modeling mice was significantly decreased after NaHS treatment (**Figure 1A**). The body weight of mice in the control group increased steadily with the days. Subsequently, after seven days of modeling with 3% DSS, compared with the control group, the body weight and colon length of mice in the model group decreased and DAI score increased significantly (**Figures 1 B–E**). Compared with normal mice, CSE knockout mice presented weight loss, shorter colon length, and higher DAI scores (p<0.01). NaHS treatment for seven days reduced weight loss, colon length decreases, and DAI scores in both the regular and CSE knockout groups (p<0.01). These results suggest that CSE-derived H_2_S ameliorates DSS-induced damage in mice with ulcerative colitis.

**Figure 2:**
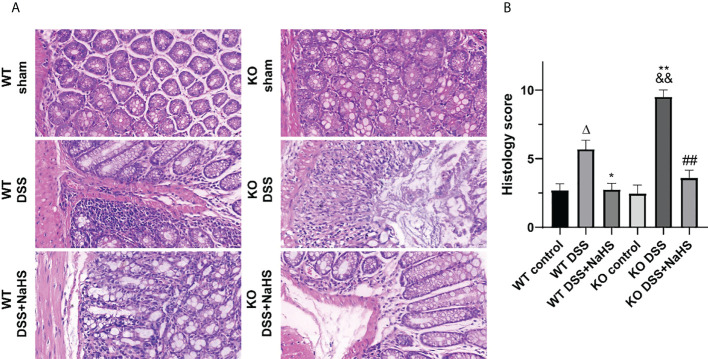
Effect of cystathionine-γ-lyase (CSE)-derived hydrogen sulfide (H2S) on colonic injury and histological score (hematoxylin and eosin (H&E) staining). Data are expressed as the mean ± SEM, N=3. Scale bar = 20μm. **(A)** Typical histological sections stained with H&E (400×magnification). **(B)** Histological scores of colon. ΔP < 0.05 vs wild-type(WT) control group; *P < 0.05, **P < 0.01 vs WT dextran sodium sulfate (DSS) group; ^&&^P < 0.01 vs knockout (KO) control group; ^##^P<0.01 vs KO DSS group.

HE staining was used to detect the pathological changes of colonic mucosa in mice with ulcerative colitis induced by DSS. The results show that the epithelial cells and crypt structure are intact, and goblet cells are intact in all the control groups. All DSS model groups had severe lesions, including loss of colonic epithelial cells, distorted crypt structure, and massive inflammatory cell infiltration. However, the damage was more severe in the CSE knockout model group than in the normal model group (**Figure 2**, p<0.01). Furthermore, compared with the DSS model group, the colon of NaHS-treated mice showed ameliorating colon injury, less inflammatory cell infiltration, and only slight crypt deformation. In addition, the histological damage score caused by DSS was significantly reduced in all NaHS treatment groups **(** **Figure 2**, p<0.01). Our results suggest that CSE-derived H_2_S significantly protects colon and attenuates DSS-induced histopathological lesions.

**Figure 3:**
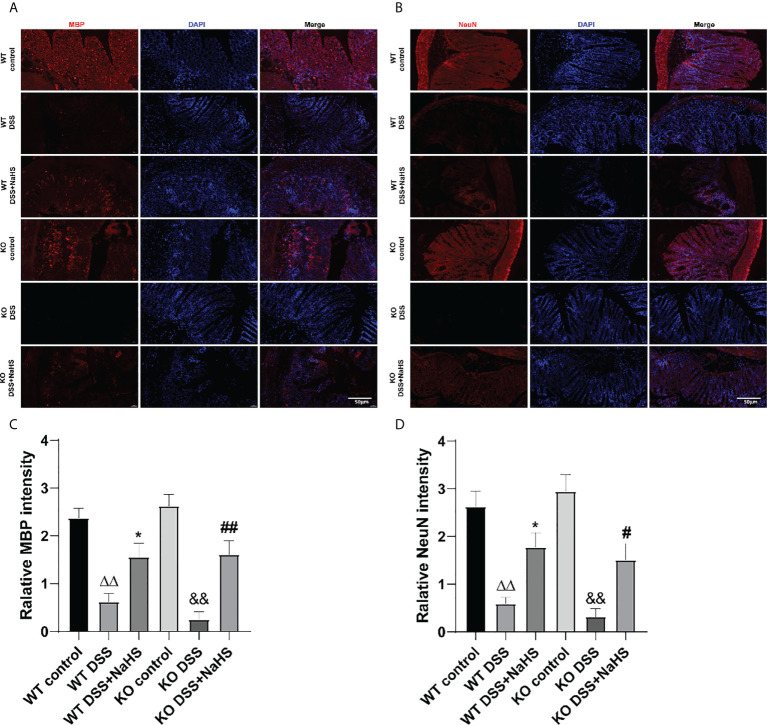
Effect of cystathionine-γ-lyase (CSE)-produced hydrogen sulfide (H2S) on the recovery of myelin sheaths and loss of neurons in colon of mice after dextran sodium sulfate (DSS). Scale bar=50μm. **(A)** Myelin basic protein (MBP)-positive cells are red, 4’,6-diamidino-2-phenylindole (DAPI) nuclear staining is blue (200×magnification). **(B)** Neuronal Nuclei (NeuN)-positive cells are red, DAPI nuclear staining is blue (200×magnification). **(C)** Relative MBP intensity in the colon of mice. **(D)** Relative NeuN intensity in the colon of mice. ΔΔP < 0.01 vs wild-type (WT) control group; *P < 0.05 vs WT dextran sodium sulfate (DSS) group; ^&&^P < 0.01 vs knockout (KO) control group; ^#^P<0.05,^##^P<0.01 vs KO DSS group.

To further investigate the effect of CSE-derived H_2_S on colon inflammation and injury, immunofluorescence (IF) staining was used to detect the loss of neuronal nucleus (NeuN) and the reduction of myelin basic protein (MBP). As shown in **Figure 3**, the fluorescence intensity of MBP and NeuN decreased after DSS modeling (compared with the control group, p<0.01), suggesting that colitis induced neuron loss and myelin sheath injury of oligodendrocytes. Furthermore, neurons and myelin sheath loss were more severe in CSE KO mice than in WT mice, and NaHS blocked this damage. These results further confirm the protective effect of CSE-derived H_2_S on neuronal injury in colon.

### CSE-derived H_2_S has a protective effect on the proliferation of EGCs

**Figure 4:**
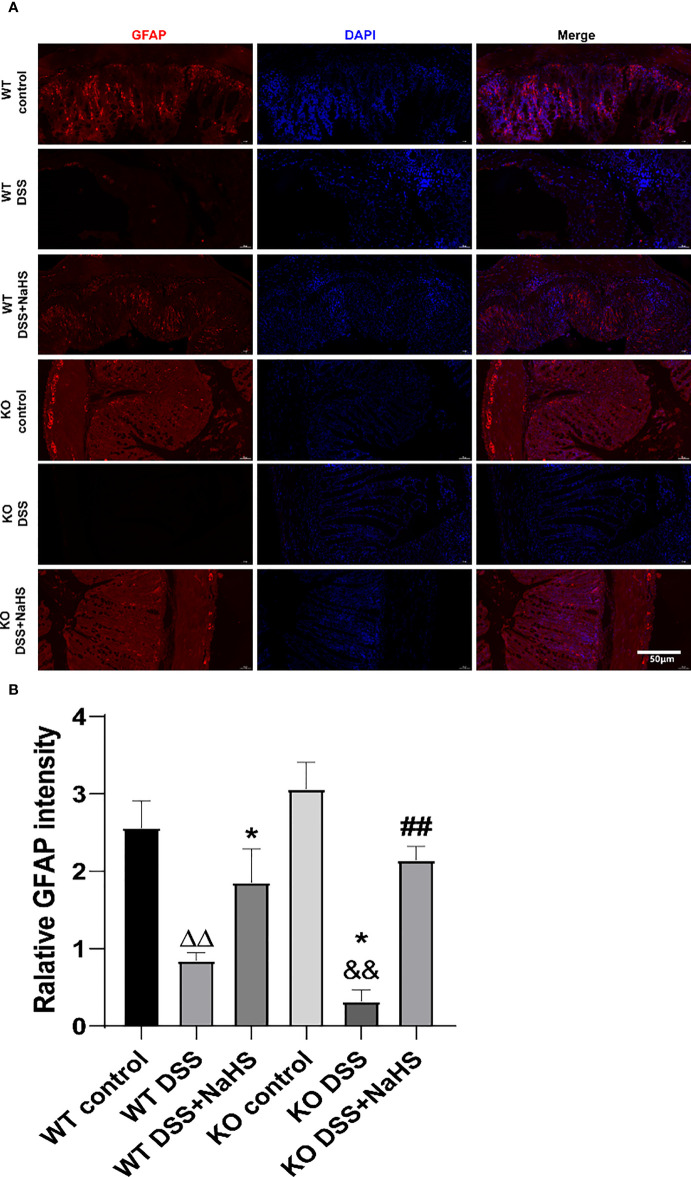
Effect of cystathionine-γ-lyase (CSE)-produced hydrogen sulfide (H_2_S) on the reactive astrocytes proliferation in colon of mice after dextran sodium sulfate (DSS) (mean±SEM, N=3). Scale bar=50μm. **(A)** glial fibrillary acidic protein (GFAP)-positive cells are red, 4’,6-diamidino-2-phenylindole (DAPI) nuclear staining is blue (200×magnification). **(B)** Relative GFAP intensity in the colon of mice.^ΔΔ^P < 0.01 vs wild-type(WT) control group; *P < 0.05 vs WT DSS group; ^&&^P < 0.01 vs knockout(KO) control group; ^##^P<0.01 vs KO DSS group.

As shown in **Figure 4**, the fluorescence intensity of GFAP decreased in colonic mucosa of mice after colitis (compared with the control group; p< 0.01). However, the fluorescence intensity of GFAP in colonic mucosa of CSE knockout mice induced by colitis was significantly decreased compared with that of WT mice. In the NaHS treatment, colitis-induced GFAP fluorescence intensity was enhanced considerably. These data suggest that H_2_S has a significant protective effect on the proliferation of astrocytes.

### CSE-derived H_2_S promoted the transformation of EGCs from type “A1” to “A2”

**Figure 5:**
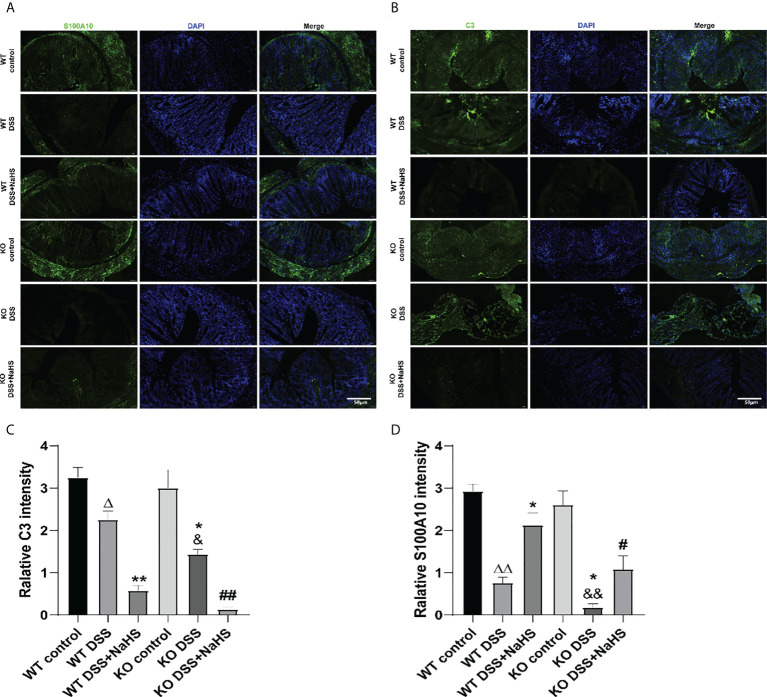
Effect of cystathionine-γ-lyase (CSE)-produced hydrogen sulfide (H_2_S) on the transformation of enteric glial cells (EGCs) from “A1” to “A2” in colon of mice after dextran sodium sulfate (DSS) (mean ± SEM, N=3). Scale bar=50μm. **(A)** S100A10-positive cells are green, 4’,6-diamidino-2-phenylindole (DAPI) nuclear staining is blue (200×magnification). **(B)** C3-positive cells are green, DAPI nuclear staining is blue (200×magnification). **(C)** Relative S100A10 intensity in the colon of mice. **(D)** Relative C3 intensity in the colon of mice. ^Δ^P < 0.05, ^ΔΔ^P < 0.01 vs wild-type(WT) control group; ^*^P < 0.05, ^**^P < 0.01 vs WT DSS group; ^&^P < 0.05, ^&&^P < 0.01 vs knockout(KO) control group; ^#^P<0.05,^##^P<0.01 vs KO DSS group.

To further study the transformation of A1/A2 reactive astrocytes in DSS mouse colon, C3 and S100A10 staining were performed. As shown in **Figure 5**, the fluorescence intensity of C3 and S100A10 in colon decreased seven days after DSS modeling. Besides, the fluorescence intensity of S100A10 and C3 in colon of CSE KO mice decreased more significantly than that of WT mice. In addition, we found that NaHS could further reduce the DSS-induced C3 fluorescence intensity and promote the increase of S100A10 fluorescence intensity in colon. These results confirm that CSE-derived H_2_S can promote the reactive proliferation of astrocytes in colitis-induced colon and promote the transformation of astrocytes from “A1” type to “A2” type.

### The effects of CSE-derived H_2_S on the activities of RhoA, ROCK_1_, and ROCK_2_ proteins and the content of H_2_S in colon, as well as the inflammatory cytokines IL-6 and TNF-α in serum of mice

**Figure 6:**
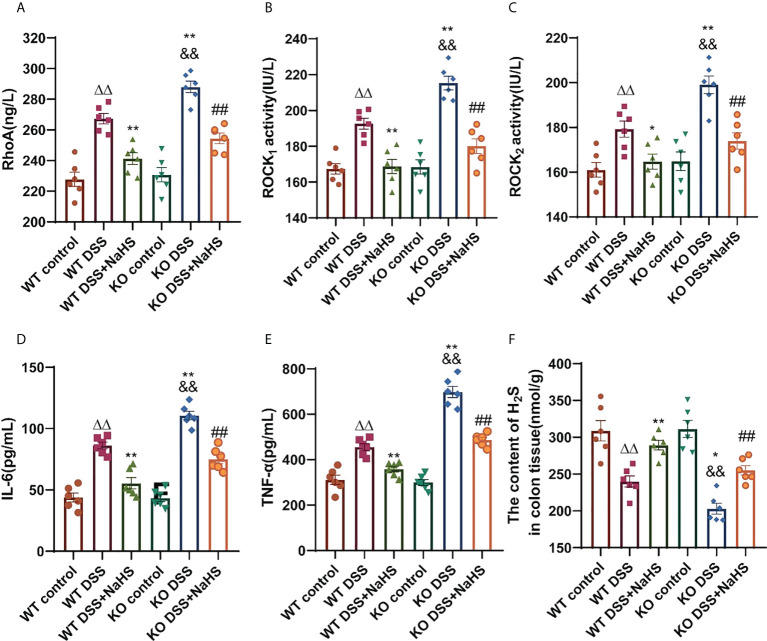
Effect of cystathionine-γ-lyase (CSE)-derived hydrogen sulfide (H_2_S) on colonic RhoA, Rho kinase 1 (ROCK_1_), Rho kinase 2 (ROCK_2_) protein activity and changes of interleukin-6 (IL-6) and tumor necrosis factor-α (TNF-α) in mice sera, and hydrogen sulfide (H_2_S) content in colon. Data are expressed as the mean ± SEM, N=6. RhoA activity **(A)**, ROCK_1_ activity **(B)**, ROCK_2_ activity **(C)**, IL-6 **(D)**, and TNF-α **(E)** in sera determined by ELISA. The concent of H_2_S in colon (nmol/g protein) determined by ELISA **(F)**. ^ΔΔ^P < 0.01 vs wild-type(WT) control group; *P < 0.05, **P < 0.01 vs WT dextran sodium sulfate (DSS) group; ^&&^P < 0.01 vs knockout (KO) control group; ^##^P<0.01 vs KO DSS group.

As shown in **Figure 6**, RhoA, ROCK_1_, ROCK_2_ protein activities, and H_2_S content in colon were detected by ELISA. Compared with the control group, the protein activities of RhoA, ROCK_1_ and ROCK_2_ were enhanced, and the content of H_2_S was decreased in the DSS model group. The enhanced RhoA, ROCK_1_, and ROCK_2_ protein activities and the reduced H_2_S content in the CSE knockout model group were significantly more substantial than those in the normal model group (**Figures 6 A–C, F**, p< 0.01). In addition, the NaHS treatment group reduced RhoA, ROCK_1_, and ROCK_2_ protein activities and significantly enhanced H_2_S content in the model group (**Figures 6 A–C, F**, p< 0.01). The inflammatory cytokines IL-6 and TNF-α in the serum of mice showed the same upregulation trend as the RhoA/ROCK signaling pathway (**Figures 6 D, E**, p< 0.01). Our results suggest that CSE-derived H_2_S protects colon and attenuates inflammatory cytokines IL-6 and TNF-α levels by decreasing RhoA, ROCK_1_, and ROCK_2_ protein activities and increasing H_2_S.

### Effect of CSE-derived H_2_S on the expression of RhoA, ROCK_1_, and ROCK_2_ proteins in colon

**Figure 7:**
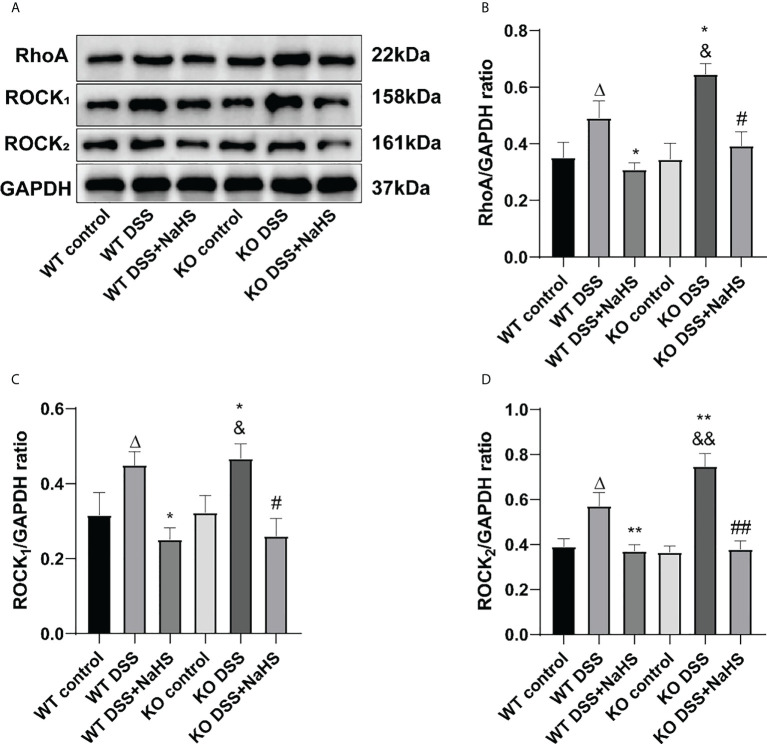
Effects of cystathionine-γ-lyase (CSE)-derived hydrogen sulfide (H_2_S) on the expression of RhoA, Rho kinase 1 (ROCK_1_), and Rho kinase 2 (ROCK_2_) proteins in colon. Data are expressed as the mean ± SEM, N=3. **(A)** The bands of RhoA, ROCK_1_, and ROCK_2_. **(B)** The relative expression of RhoA in colon. **(C)** The relative expression of ROCK_1_ in colon. **(D)** The relative expression of ROCK_2_ in colon. ^Δ^P < 0.05 vs wild-type(WT) control group; *P < 0.05，**P < 0.01 vs WT dextran sodium sulfate (DSS) group; ^&^P < 0.05, ^&&^P < 0.01 vs knockout (KO) control group; ^#^P<0.05, ^##^P<0.01 vs KO DSS group.

To clarify the correlation between CSE-derived H_2_S and RhoA/ROCK pathway, Western blot was used to detect the expression of the RhoA/ROCK pathway. The terms RhoA, ROCK_1_, and ROCK_2_ detected by Western blot are shown in **Figure 7**. The expression of RhoA, ROCK_1_ and ROCK_2_ was significantly increased in colitis. Therefore, it is not surprising that colitis-induced RhoA, ROCK_1_, and ROCK_2_ expression levels were even higher in CSE KO mice than in WT mice. However, continuous treatment with NaHS from day one after colitis decreased the expression of RhoA, ROCK_1_, and ROCK_2_ proteins in CSE KO and WT mice. These data suggest that CSE-derived H_2_S inhibits the activation of the RhoA/ROCK pathway.

### Effect of CSE-derived H_2_S on the expression of GFAP, C3, and S100A10 proteins in colon

**Figure 8:**
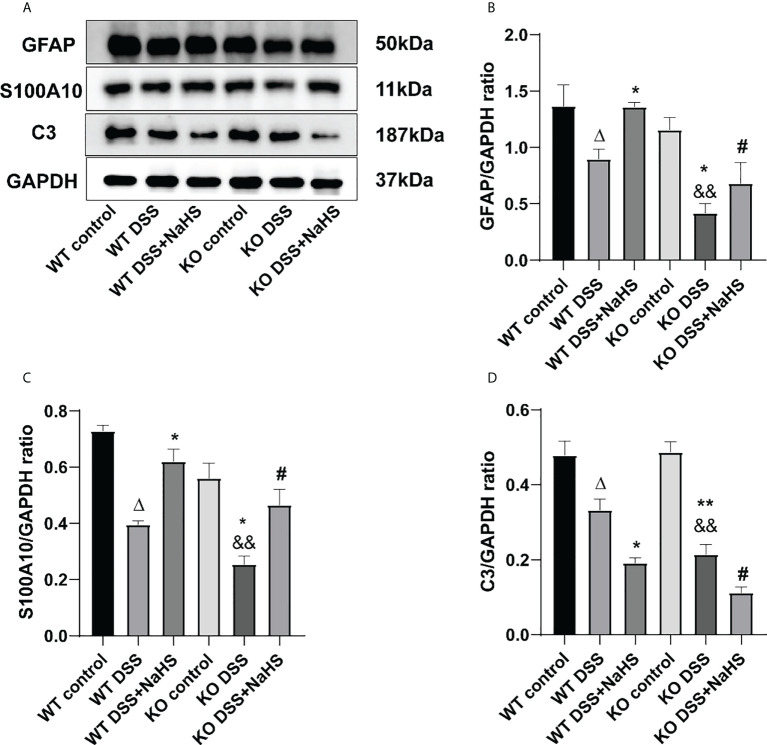
Effects of cystathionine-γ-lyase (CSE)-derived hydrogen sulfide (H2S) on the expression of glial fibrillary acidic protein (GFAP), S100A10, and C3 proteins in colon. Data are expressed as the mean ± SEM, N=3. **(A)** The bands of GFAP, S100A10, and C3. **(B)** The relative expression of GFAP in colon. **(C)** The relative expression of S100A10 in colon. **(D)** The relative expression of C3 in colon. ^Δ^P < 0.05 vs wild-type (WT) control group; *P < 0.05, **P < 0.01 vs WT dextran sodium sulfate (DSS) group; ^&&^P < 0.01 vs knockout (KO) control group; ^#^P < 0.05 vs KO DSS group.

We attempted to test the expression of GFAP, A1 astrocyte labeled C3 protein, and A2 astrocyte labeled S100A10 protein in the colon by Western blot to investigate whether H_2_S produced by CSE affected the proliferation of reactive astrocytes containing A1 and A2 types. The results in **Figure 8** indicate that GFAP expression and C3 protein and S100A10 expression are decreased in the colonic mucosa of mice after colitis. In addition, colitis-induced decreases in GFAP, C3 protein, and S100A10 expression were more significant in CSE KO mice than in WT mice. However, in WT and CSE KO mice, there were no significant differences in GFAP, C3, and S100A10 protein expression, indicating that CSE KO had no effect on GFAP, C3, and S100A10 protein expression. Still, colitis exacerbated the decrease of GFAP, C3, and S100A10 protein expression. In addition, as shown in the figure, NaHS inhibits the decreased expression of GFAP and S100A10 but further inhibits the expression of C3, suggesting that H_2_S produced by CSE inhibits the proliferation of reactive astrocytes of the type A1, but enhances the proliferation of type A2 astrocytes in mouse colon after induction colitis.

## Discussion

In the present study, the DSS-induced colitis was ameliorated by the H_2_S donor NaHS, which was manifested by improved clinical parameters and decreased serum levels of IL-6 and TNF-α. More severe changes in IL-6 and TNF-α, and reduced H_2_S content in colon were observed in CSE knockout mice. Macrophages in the colon will trigger and exacerbate DSS-induced colitis by releasing cytokines, such as IL-6, TNF-α, and IL-1β (20). It has been reported that H_2_S has an anti-inflammatory effect, which may inhibit chromatin openness at the IL-6 and TNF-α promoters by suppressing histone acetylation (21, 22). NaHS can only partially ameliorate the more malignant colitis in CSE knockout mice, which indicates that CSE-derived endogenous H_2_S plays a crucial protective effect against DSS-induced colitis.

H_2_S is produced throughout the gastrointestinal tract and helps maintain mucosal integrity (23). H_2_S may have pro-inflammatory or anti-inflammatory effects depending on the dosage and route of administration (24). Subcutaneous injection of low-dose NaHS (~5.6mg/kg) showed an anti-inflammatory effect in the C57BL6/J sepsis model induced by cecal ligation and puncture (CLP) (25). However, Zhang et al. found the pro-inflammatory effect of H_2_S donor NaHS (10mg/kg i.p.) on CLP-induced sepsis and an inhibitor of CSE [DL-propargylglycine (PAG; 50 mg/kg i.p.)], which could reverse this effect (26). DSS-induced colitis in mice mimics clinical symptoms and histopathological features in human UC, with weight loss, diarrhea, and occult blood in stools (27). In this study, more serious clinical manifestations such as DAI scores, colon length, weight loss, fecal occult blood, and histopathological features of colon were observed in CSE knockout mice. Exogenous H_2_S can relieve signs and symptoms of DSS-induced UC, with reduced disease activity index (DAI) scores, improved colon length shortening, improved histopathological inflammation, and reduced body weight loss (16, 28, 29). However, the effect and mechanism of endogenous H_2_S on colitis remain unclear. CSE/H_2_S regulates circadian-clock genes and maintains cellular glutathione biosynthesis and glucose homeostasis in C2C12 myotubes (30, 31). In the present study, CSE knockout mice were more susceptible to DSS-induced colitis than wild-type mice, which was consistent with the previous report, indicating that CSE-derived endogenous H_2_S plays a crucial protective effect against DSS-induced colitis (32).

Our findings show that RhoA/ROCK signal pathway is upregulated in colitis mice. Not surprisingly, the upregulation of the RhoA/ROCK pathway is more remarkable in CSE KO mice. NaHS serves as an effective UC treatment *via* inhibiting RhoA/ROCK signal pathway. The RhoA/ROCK (Rho-kinase) signaling pathway has been demonstrated to play an essential role in regulating biological pathways, such as those that impact smooth muscle tension levels and different kinds of physiological characteristics associated with actin cytoskeletal changes, including migration, cell adhesion, contraction, and motility (33). RhoA/ROCK_1_ dependent actomyosin contractility was increased due to cortactin deficiency, a critical regulator of intestinal epithelial barrier functions, causing altered molecular composition of epithelial junctions and increased intestinal epithelial permeability (34). H_2_S protects the central nervous system (CNS) by acting as an antioxidant and regulates cell signaling (35). It has been reported that CSE-derived H_2_S could inhibit the reactive proliferation of astrocytes and promote the recovery of neural functional deficits in mice induced by cerebral I/R injury *via* inhibition of the RhoA/ROCK_2_ signal pathway (17). However, it remains unclear whether CSE-derived H_2_S can play a similar role in the DSS-induced colitis model. In the present study, our findings indicate that CSE-derived H_2_S can promote the recovery of mice gut functional deficits from DSS-induced colitis *via* inhibition of the RhoA/ROCK signal pathway.

The enteric nervous system consists of neurons and EGCs. Compelling evidence demonstrated that enteric glia played a central role in regulating homeostasis in the enteric nervous system (36, 37). Active signaling mechanisms between enteric glia and neurons modulate gastrointestinal reflexes. In certain circumstances, its function drives the neuroinflammatory process leading to long-term dysfunction. Bidirectional communication between enteric glia and immune cells contributes to gastrointestinal immune homeostasis (36). The present study was designed to deeply elucidate the pathological changes of intestinal nerve cells in UC and determine whether the effects of endogenous H_2_S on alleviating UC were associated with improved functional defects of EGCs. Although enteric glial cells are extensive heterogeneity and phenotypic plasticity, the majority of glia in the myenteric plexus are GFAP positive (38). GFAP gene and protein expression have been a useful marker for assessing astrocyte reactivity in animal injury and disease models and in human pathological specimens (39). Our findings show that GFAP expression indicative of reactive glia is reduced in the DSS-induced colitis mice and worse in the CSE knockout mice. NaHS significantly increased GFAP protein levels during inflammation, and we observed corresponding changes in the GFAP Immunofluorescence assay. CSE-derived endogenous H_2_S had a significant effect on preventing the reactiveness of glial processes during colitis.

Enteric glia secretes neuroprotective compounds and possesses neuroprotective properties (40). Intestinal inflammation is associated with the leukocyte infiltration into or surrounding the neuronal ganglia of the enteric nervous system (ENS), which is termed plexitis or ganglionitis (41). In this study, the staining results of NeuN and MBP in the colon indicate DSS-induced neuron damage and myelin loss of glial cells in the mice colitis model. NeuN is a neuronal-specific nuclear protein that serves as an excellent marker for neurons in the central and peripheral nervous systems of vertebrate embryos and adults (42). Nassauw et al. reported that the cytoplasmic expression of NeuN is an exclusive marker of intrinsic primary afferent neurons (IPANs) in the gut (43). MBP is a membrane actin-binding protein that participates in the transmission of extracellular signals to the cytoskeleton in oligodendrocytes and tight junctions in myelin (44). Our results showed that CSE-derived endogenous H_2_S had an effect on protecting the intestinal neurons and glial cells during colitis.

In this study, we found that C3 and S100A10 protein expression were reduced in colon of DSS-induced colitis mice, which were lower in that of CSE knockout mice. NaHS treatment markedly blocked the decrease of S100A10 expression, but further reduced C3 expression. The same results were confirmed by immunofluorescence assay. Neuroinflammation and ischemia-induced two different phenotypes of reactive astrocytes, which are termed A1 and A2, in analogy to the M1/M2 macrophage nomenclature (39, 45). The upregulation of H_2_S levels can have beneficial effects on glucose homeostasis through activation of PGC-1α/FNDC5/irisin signaling pathway (46). The anti-inflammatory mechanism of irisin includes reducing macrophage proliferation and inducing alternating activation (M2 type) macrophage polarization (47). Recent studies demonstrated that different initiating CNS injuries could elicit two types of “reactive” astrocytes with different properties, type A1 is harmful and type A2 is beneficial or with reparative functions (39). Complement component C3 is one of the most characteristic and highly upregulated genes in A1s but is not expressed in A2 reactive astrocytes, while S100A10 is specifically expressed in the A2 type astrocytes (45, 48). To our knowledge, the heterogeneity of reactive astrocytes in the gut has not been reported and requires further study. Our previous study showed that the number of astrocytes in the DSS-induced colitis colon was decreased and H_2_S had an inhibitory effect on the decrease and promoted the transformation of the A1 type into the A2 type (16). The present results indicate that CSE-derived endogenous H_2_S plays an important role in protecting colitis by promoting the proliferation of EGCs, suppressing the A1 phenotype conversion in astrocytes and promoting the transformation of astrocytes from “A1” type to “A2” type. These findings suggest that preserving the loss of EGCs or transforming the pathologic astrocytes into useful ones can reverse ENS dysfunction.

In conclusion, these findings indicate that CSE-derived H_2_S can preserve the function of EGCs *via* inhibiting the reactive proliferation and promote EGCs transformation, and inhibition of the RhoA/ROCK signal pathway in mice model of colitis induced by DSS.

## Data availability statement

The raw data supporting the conclusions of this article will be made available by the authors, without undue reservation.

## Ethics statement

The animal study was reviewed and approved by Animal Experimental Ethics Review Committee of the University of Science and Technology of China.

## Author contributions

SW and WJ conceived the ideas and designed the study. SW and YD performed laboratory work. All authors analyzed data. SW and WJ wrote the paper and the manuscript was reviewed and commented on by all authors. All authors contributed to the article and approved the submitted version.

## Acknowledgments

We are grateful to Dr. Xudong Yuan and Dr. Yiyao Tu for the colon pathological analysis.

## Conflict of interest

The authors declare that the research was conducted in the absence of any commercial or financial relationships that could be construed as a potential conflict of interest.

## Publisher’s note

All claims expressed in this article are solely those of the authors and do not necessarily represent those of their affiliated organizations, or those of the publisher, the editors and the reviewers. Any product that may be evaluated in this article, or claim that may be made by its manufacturer, is not guaranteed or endorsed by the publisher.
